# Metabolism and Interspecies Variation of IMMH-010, a Programmed Cell Death Ligand 1 Inhibitor Prodrug

**DOI:** 10.3390/pharmaceutics13050598

**Published:** 2021-04-21

**Authors:** Yuchen Wang, Xiao Liu, Xiaowen Zou, Shuting Wang, Lijun Luo, Yuke Liu, Kai Dong, Xiaoqing Yao, Yan Li, Xiaoguang Chen, Li Sheng

**Affiliations:** 1 Beijing Key Laboratory of Active Substances Discovery and Druggability Evaluation, Institute of Materia Medica, Chinese Academy of Medical Sciences and Peking Union Medical College, 1 Xian Nong Tan Street, Beijing 100050, China; wangyuchen@imm.ac.cn; 2 State Key Laboratory of Bioactive Substance and Function of Natural Medicines, Institute of Materia Medica, Chinese Academy of Medical Sciences and Peking Union Medical College, 1 Xian Nong Tan Street, Beijing 100050, China; liuxiao@imm.ac.cn (X.L.); zouxiaowen@imm.ac.cn (X.Z.); wangshuting@imm.ac.cn (S.W.); luolijun@imm.ac.cn (L.L.); liuyuke@imm.ac.cn (Y.L.); yanli@imm.ac.cn (Y.L.); 3 Beijing Key Laboratory of Non-Clinical Drug Metabolism and PK/PD Study, Institute of Materia Medica, Chinese Academy of Medical Sciences and Peking Union Medical College, 1 Xian Nong Tan Street, Beijing 100050, China; 4 Department of Drug Metabolism, Institute of Materia Medica, Chinese Academy of Medical Sciences and Peking Union Medical College, 1 Xian Nong Tan Street, Beijing 100050, China; 5 Tianjin Chase Sun Pharmaceutical Co. Ltd., 20 Quanfa Road, Tianjin Wuqing Development Area, Tianjin 300170, China; dongkai@chasesun.cn (K.D.); yaoxiaoqing@chasesun.cn (X.Y.)

**Keywords:** PD-1/PD-L1 inhibitor, intestinal metabolism, carboxylesterase 1, drug metabolism, prodrug

## Abstract

IMMH-010 is an ester prodrug of YPD-29B, a potent programmed cell death ligand 1 (PD-L1) inhibitor. The metabolism of IMMH-010 was investigated and compared in various species. Four metabolites of IMMH-010 were identified, and the major metabolite was the parent compound, YPD-29B, which was mainly catalyzed by carboxylesterase 1 (CES1). We observed IMMH-010 metabolism in the plasma of various species. IMMH-010 was rapidly metabolized to YPD-29B in rat and mouse plasma, whereas it remained stable in human and monkey plasma. In the liver S9 fractions of human, monkey, dog, and rat, IMMH-010 was quickly transformed to YPD-29B with no obvious differences among species. In addition, the transformation ratio of IMMH-010 to YPD-29B was low in rat and human intestines, which indicated that the intestine was not an important site for IMMH-010 hydrolysis. Moreover, we demonstrated the remarkable antitumor efficacy of IMMH-010 in B16F10 melanoma and MC38 colon carcinoma xenograft mouse models. We also compared the pharmacokinetic profiles of IMMH-010 in rodents and primates. After oral administration of IMMH-010, the general exposure of active metabolite YPD-29B was slightly lower in primates than in rodents, suggesting that data should be extrapolated cautiously from rodents to humans.

## 1. Introduction

Programmed cell death protein 1 (PD-1) is a cell surface receptor usually expressed on activated T cells [1]. Programmed cell death ligand 1 (PD-L1) is overexpressed in various cancerous cells, and overexpressed PD-L1 often binds with PD-1 and inhibits the antitumor immune response mediated by T cells, eventually causing the immune evasion of tumors [2,3]. PD-1/PD-L1 checkpoint pathway blocking is an effective immunotherapy strategy for cancer treatment [4]. In the past decade, several specific PD-1/PD-L1 antibodies have been developed and marketed as anti-cancer drugs that have achieved spectacular success in anti-cancer treatments. Moreover, the number of anti-PD-1/PD-L1 antibodies is still rapidly increasing, and they are clinically proven to treat various malignancies [5]. However, antibody-derived drugs usually have inherent disadvantages, such as the need for intravenous injections, complex production, instability, high cost, low penetration into tissues, and immune-related adverse events [6,7]. Thus, it is desirable to develop small-molecule PD-1/PD-L1 inhibitors, which are expected to overcome the problems with antibody-derived drugs but retain the desirable anti-cancer efficacy [8].

**Figure 1:**
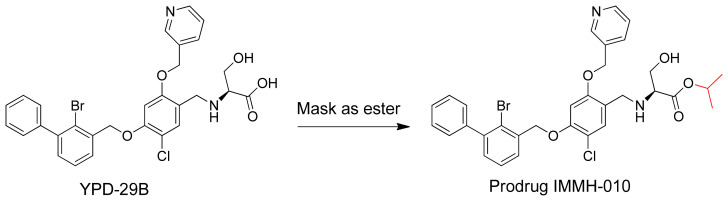
Modification of potent PD-L1 inhibitor YPD-29B to ester prodrug IMMH-010 in order to resolve the undruggable physiochemical properties.

Since the crystal structure of the human PD-1/PD-L1 complex was first published in 2015, several small-molecule PD-1/PD-L1 inhibitors with potent inhibitory activities have been reported [9]. YPD-29B blocks the binding of PD-1 and PD-L1 with an IC_50_ of less than 10^−13^ M (patent: CN109153670), measured using a homogeneous time-resolved fluorescence protein-protein interaction assay. However, during drug chemistry, manufacturing, and controls (CMC), YPD-29B encountered intractable problems. Thus, the carboxylic acid of YPD-29B was masked, giving the ester prodrug IMMH-010 (Figure 1). IMMH-010 is expected to be hydrolyzed efficiently to produce YPD-29B as the active metabolite in vivo. In addition, IMMH-010 exhibited some PD-L1 inhibitory activity with an IC_50_ of 10^−8^–10^−7^ M. Currently, IMMH-010 is in phase I clinical trials [10].

Although the prodrug strategy can improve the druggability of some compounds, it is necessary to investigate prodrug metabolism to determine whether the parent compound is released in vivo as desired. In addition, mouse models are widely used in various pharmacology studies, particularly in anti-cancer research. However, there are differences in metabolism among species. For example, there are species and tissue differences in esterase activities that cause species differences in hydrolase activity and efficacy [11]. Thus, prodrug metabolism should also be investigated in different species, particularly the pharmacokinetics (PK)/pharmacodynamics (PD) relationship. These data provide important information for the drug CMC development and for clinical applications.

In this study, we clarify the metabolic pathway of IMMH-010, compare the differences in IMMH-010 metabolism among species in vivo and in vitro, and analyze the in vivo PK/PD relationship of IMMH-010 in tumor-bearing mice.

## 2. Materials and Methods

### 2.1. Chemicals and Reagents

IMMH-010, YPD-29B (M1, purity ≥99%), M2, M3, and M4 (purity ≥98%) were synthesized by the Department of Medicinal Chemistry (Institute of Materia Medica, Chinese Academy of Medical Sciences). β-Nicotinamide adenine dinucleotide phosphate (NADP), glucose-6-phosphate, glucose-6-phosphate dehydrogenase, midazolam, telmisartan, and phenacetin were obtained from Sigma Aldrich (St. Louis, MO, USA). Lidocaine and CPT-11 were purchased from J&K Scientific Ltd. (Beijing, China). Male mouse and rat liver microsomes and liver S9 homogenate fractions were prepared by our group. Pooled mixed-gender human liver microsomes (HLM); male beagle dog and monkey liver microsomes; pooled mixed-gender dog, monkey, and human liver S9 homogenate fractions; recombinant human cytochrome P450s (CYPs); and recombinant human flavin-containing monooxygenases (FMOs) were obtained from BD Gentest (Woburn, MA, USA). HPLC-grade acetonitrile and methanol were purchased from Merck (Darmstadt, Germany). Deionized water was obtained from a Milli-Q system (Millipore Co., Billerica, MA, USA). All other reagents and solvents were commercially available. Recombinant human carboxylesterases (CESs; CES1 and CES2) were purchased from Cypex Ltd. (Dundee, United Kingdom), and recombinant human arylacetamide deacetylase (AADAC) was purchased from CUSABIO Biotech Co., Ltd. (Wuhan, China).

### 2.2. Animals

Eight-week-old male C57BL/6 mice and six-week-old male Sprague-Dawley rats were purchased from Beijing Vital River Laboratory Animal Technology Co., Ltd. (Beijing, China). Standard pelleted laboratory chow and water were allowed ad libitum. The animal protocol followed was approved by the Experimental Animal Management and Welfare Committee at the Institute of Materia Medica, Peking Union Medical College. Healthy male cynomolgus monkeys (3–4 years old, approximately 3 kg) were purchased from Guangdong Frontier Biotechnology Co., Ltd. (Guangzhou, China). The animal protocol was approved by the Animal Experimental Ethics Committee of Joinn Laboratories (Joinn Laboratories, Inc., Beijing, China) (No.ACU18-324) and the Experimental Animal Management and Welfare Committee at the Institute of Materia Medica, Peking Union Medical College (No. 00000522, No. 00000523, No. 00000538, No. 00000539).

### 2.3. Identification of IMMH-010 Metabolites

After oral administration of IMMH-010 maleate (5 mg/kg), two rats were housed in metabolic cages to collect urine and feces samples for 24 h. Feces samples were homogenized with methanol at 1:10 (*w*/*v*) and urine samples were diluted 10-fold in methanol. Additionally, two rats were bile duct cannulated under isoflurane anesthesia. After oral administration of IMMH-010 maleate (5 mg/kg), bile samples were collected over 0–2 h after dosing.

LC-MS was performed using a Thermo Scientific Q Exactive HF hybrid quadrupole-Orbitrap mass spectrometer. Reverse-phase liquid chromatography was performed on a Zorbax C18 column (100 × 2.1 mm, 3.5 μm). Mobile phase A was 0.1% aqueous formic acid and mobile phase B was 0.1% formic acid in methanol. The mobile phase was delivered with the following gradient elution profile: 0 min, 10% B; 15 min, 40% B; 17–19 min, 100% B; 20–23 min, 10% B. The total runtime for each injection was 23 min. The flow rate was 0.3 mL/min, and the column oven was maintained at 30 °C.

The analysis was performed in full mass spectrometry with data-dependent tandem mass spectrometry (MS/dd-MS^2^) mode. For a full mass spectrometry scan, the selected scan range was from *m*/*z* 100 to 500, the resolution was 70,000, and the automatic gain control (AGC) target was set to 1.0 × 10^6^ with a maximum injection time (IT) of 100 ms. For the dd-MS^2^ scan, the fragmentation mass spectra were recorded at a mass resolving power of 17,500 fwhm with a quadrupole isolation window of 1.5 *m*/*z* for precursor ions. The AGC target was 2.0 × 10^5^ and the maximum IT was 50 ms.

Xcalibur 4.1 software (Thermo Fisher Scientific, San Jose, CA, USA) was used for instrument control and data processing. Compound Discover 2.0 software was used for metabolite identification.

### 2.4. IMMH-010 Metabolism in Plasma, S9 Fractions and Microsomes from Liver and Intestine

IMMH-010 hydrolysis was investigated in human, monkey, rat, and mouse plasma. IMMH-010 (1 μM) was incubated in human, monkey, or dog plasma (0.2 mL) at 37 °C for 120 min. IMMH-010 (10 μM) was incubated in rat or mouse plasma (0.2 mL) at 4 °C for 60 min. The experiments were performed in triplicate. The reactions were initiated by the addition of IMMH-010 (10 μM). After incubation, the reactions were stopped by adding 2 volumes of ice-cold acetonitrile. After vortexing and centrifuging, the concentrations of IMMH-010 and YPD-29B in the supernatant were determined by liquid chromatography with tandem mass spectrometry (LC-MS/MS). The compound olmesartan (10 μM), a substrate of paraoxonase 1, was set as the positive control.

IMMH-010 metabolism was compared in human, monkey, dog, and rat liver S9 homogenates. IMMH-010 (10 μM) was incubated with liver S9 homogenate protein (0.5 mg protein/mL) and a reduced nicotinamide adenine dinucleotide phosphate (NADPH) regenerating system in a final volume of 0.2 mL Tris-HCl buffer (50 mM, pH 7.4) containing 5 mM MgCl_2_. The reactions were performed in triplicate. After 2 min of preincubation at 37 °C, reactions were initiated by the addition of IMMH-010 (10 μM). The samples were collected 0, 5, 10, and 20 min after incubation, 2 volumes of acetonitrile were added to terminate the reaction. After vortexing and centrifugation, IMMH-010, YPD-29B, M3, and M4 in the supernatant were analyzed by LC-MS/MS.

To compare the differences in IMMH-010 metabolism between the liver and intestine, IMMH-010 (10 μM) was incubated with rat liver and intestinal S9 homogenate protein (1 mg protein/mL) and with human liver and intestinal microsomes (0.2 mg protein/mL) in a final volume of 0.2 mL Tris-HCl buffer (50 mM, pH 7.4) containing 5 mM MgCl_2_. The incubations were performed in duplicate in the presence and absence of an NADPH regenerating system. After 2 min preincubation at 37 °C, reactions were initiated by the addition of IMMH-010 (10 μM). Incubations were terminated by acetonitrile at 0, 10, and 20 min. IMMH-010, YPD-29B, M2, and M3 were quantified by LC-MS/MS assay. Midazolam (10 μM, a substrate of CYP3A4) was set as the positive control.

### 2.5. Identification of Metabolizing Enzymes

Selective chemical inhibitors and recombinant human enzymes were used to investigate the esterase responsible for IMMH-010 metabolism in HLM. The selective CES1 and CES2 inhibitors were digitonin (100 μM) and telmisartan (50 μM), respectively. IMMH-010 (10 µM) was incubated with HLM (0.2 mg/mL) at 37 °C for 0 and 15 min in the presence of chemical inhibitors. Control samples were incubated without inhibitors.

To verify the role of CES1, CES2, and AADAC in human metabolism of IMMH-010, IMMH-010 (10 µM) was incubated individually with recombinant human CES1, CES2, and AADAC (0.1 mg protein/mL) at 37 °C for 15 min. Lidocaine (500 μM), CPT-11 (2 μM), and phenacetin (1 mM), which are substrates of CES1, CES2, and AADAC, were used as positive controls.

cDNA-expressed human CYPs were also used to investigate the enzymes mediating IMMH-010 metabolism. IMMH-010 (10 µM) was incubated individually with 50 pmol of 10 individual cDNA-expressed human CYPs (CYP1A1, CYP1A2, CYP2A6, CYP2B6, CYP2C9, CYP2C19, CYP2D6, CYP2E1, CYP2J2, or CYP3A4) and three cDNA-expressed human FMO enzymes (FMO1, FMO3, and FMO5) at 37 °C for 30 min. An NADPH regeneration system was added to initiate the reactions. Probe substrates of the CYPs were used as the positive controls. Incubations without NADPH served as the negative controls. All reactions were carried out in a final volume of 0.2 mL Tris-HCl buffer (50 mM, pH 7.4) containing 5 mM MgCl_2_ and conducted in triplicate. All incubations were terminated by 2 volumes of acetonitrile. After centrifugation at 14,000 rpm for 5 min, the samples were analyzed by LC-MS/MS.

### 2.6. PK/PD Study in Mice

The antitumor activity of IMMH-010 was evaluated by B16F10 melanoma and MC38 colon carcinoma xenograft models. The B16F10 and MC38 cells were resuspended in saline (1.5 × 10^6^ cells/0.2 mL saline) and injected subcutaneously into the right flanks of each mouse on day 0. The treated mice received once-daily oral administration of IMMH-010 maleate (1.25, 2.5, 5, and 10 mg/kg, *n* = 10) by oral gavage (PO) for 19 consecutive days, whereas the control mice received vehicle (0.5% carboxymethyl cellulose, *n* = 10). The mice in the positive control group received the anti-mouse PD-L1 antibody (696618M2, Bio X Cell, West Lebanon, NH, USA) at 10 mg/kg intraperitoneally every 3 days (*n* = 10). The first-line antineoplastic drug cyclophosphamide (CTX) was administered at doses of 80 and 40 mg/kg weekly in the B16F10 and MC38 models (*n* = 10), respectively [12]. After the last treatment, on day 19, the mice were sacrificed, and the tumor and spleen were stripped and weighed. The tumor growth inhibition (TGI) was calculated as TGI = (1 − treatment group tumor weight/vehicle group tumor weight) × 100.

After the final dose, tumor and blood samples treated with IMMH-010 maleate (5 mg/kg) were collected over 72 h. Blood was exposed in an EP tube containing 500 mM NaF and 0.5% heparin sodium. Plasma samples were obtained by centrifugation, and tumor tissues were homogenized with 3 volumes (*w*/*v*) of saline on ice. The concentration of IMMH-010 and YPD-29B in plasma and tumor samples was quantitated by an LC-MS/MS method.

### 2.7. PK Study in Monkeys

IMMH-010 maleate was suspended with 0.5% carboxymethyl cellulose to make a 1 mg/mL suspension for PO gavage. Four male cynomolgus monkeys received a single oral dose of IMMH-010 maleate (5 mg/kg). Serial blood samples were collected upto 48 h. The plasma was separated and the plasma concentrations of IMMH-010 and YPD-29B were determined by LC-MS/MS.

### 2.8. LC-MS/MS Analysis

LC-MS/MS was performed using a triple quadrupole mass spectrometer (API 4000, AB Sciex, Framingham, MA, USA) with an ultra-performance liquid chromatography system (LC-30A, Shimadzu, Kyoto, Japan). Analyst 1.6.2 software (AB Sciex) was used for data acquisition and analysis. Mobile phase A was 1 mM ammonium acetate and mobile phase B was methanol. The mobile phase was delivered with the following gradient elution profile: 0–2.8 min, 70% B; 2.9 min, 85% B; 3 min, 90% B; 3.1–6.8 min, 100% B; 6.9–12 min, 70% B. The flow rate was 0.3 mL/min, and the column oven was maintained at 35 °C. The specific transitions monitored were 641.3 → 494.2 for IMMH-010, 597 → 155 for YPD-29B, 512 → 247 for M3, and 524 → 246 for M4.

### 2.9. Data Analysis

The apparent permeability coefficient (P_app_) was determined according to the following equation: P_app_ = dQ/dt × (1/AC_0_), where dQ/dt is the permeability rate, A is the area of the inserts, and C_0_ is the initial concentration. The efflux ratio was defined as the ratio of P_app_ in the basolateral-to-apical direction divided by P_app_ in the apical-to-basolateral direction.

All statistics were calculated using GraphPad Prism 8.0 software (San Diego, CA, USA) designed for one-way ANOVA. Pharmacokinetic parameters were calculated with a non-compartmental analysis using WinNonlin Version 6.3 (Pharsight, Mountain View, CA, USA). ADMET predictor V10.0 (Simulations Plus, Inc., Lancaster, CA, USA) and Microsoft Excel (Microsoft, Redmond, WA, USA) were used to process the data (i.e., half-life (t_1/2_) and hepatic clearance (CL_hep_) determination). Data were expressed as means ± standard deviations (SD). Results were considered statistically significant if the *p-*value <0.05, <0.01 and <0.001.

## 3. Results

### 3.1. Identification of IMMH-010 Metabolites

**Figure 2:**
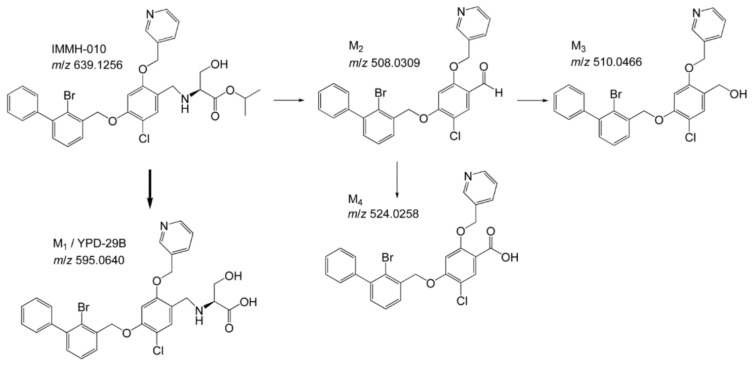
Metabolites of IMMH-010. The predominant metabolite is YPD-29B. M2, M3, and M4 are the IMMH-010 metabolites in which serine is removed, consistent with the synthesized reference compounds.

The metabolic profiles of IMMH-010 in rat urine, bile, and feces were analyzed by LC-MS/MS in MS/dd-MS^2^ mode. Four major IMMH-010 metabolites (YPD-29B, M2, M3, and M4) were detected in rat feces and bile. Only YPD-29B was found in urine. IMMH-010, YPD-29B, M2, M3, and M4 were eluted at 8.15, 7.94, 9.40, 8.85, and 9.14 min, respectively. YPD-29B had the [M − H]^−^ ion at *m*/*z* 595.0640 in the full-scan experiment, corresponding to the loss of the isopropyl group. M2, M3, and M4 exhibited the [M + H]^+^ ion at *m*/*z* 508.03096, 510.04661, and 524.02587, respectively. The major fragment ions of IMMH-010 were 244.995, 166.077, 492.035, and 336.037. M2 produced three main fragment ions at *m*/*z* 244.995, 467.349, and 166.077. M3 showed fragment ions at *m*/*z* 244.995 and 166.177. The chief fragment ions of M4 were 244.995, 166.077, and 337.044. Since M2, M3, and M4 shared the same fragment ions at *m*/*z* 166.077 and 244.995, suggesting that they may all have had changes in the serine side chain. Because the retention times and fragmentation profiles were consistent with the synthesized reference compounds, M2, M3, and M4 were identified as the IMMH-010 metabolites in which serine is removed (Figure 2).

### 3.2. Metabolism of IMMH-010 in Plasma

**Figure 3:**
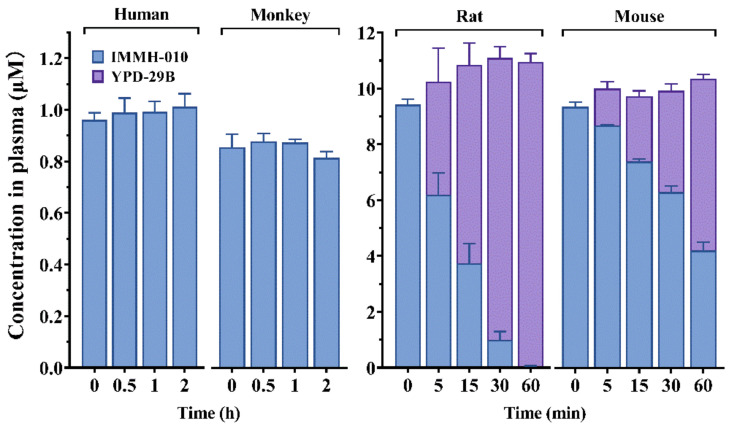
In vitro stability of IMMH-010 in human, monkey, rat, and mouse plasma. IMMH-010 decreased time-dependently in rat and mouse plasma, and the amount of YPD-29B generated was close to the amount by which IMMH-010 decreased. Data are expressed as mean ± SD.

The plasma stability was evaluated in human, monkey, rat, and mouse plasma using olmesartan, a substrate of paraoxonase 1, as the positive control. After 1 h of incubation, the control compound was decreased by more than 80% in all four kinds of plasma, indicating that the incubation systems were active and reliable. Then, we evaluated the plasma stability of IMMH-010 (Figure 3). After 2 h of incubation at 37 °C, no active metabolite YPD-29B was observed in monkey and human plasma. In contrast, despite being kept at 4 °C, IMMH-010 decreased time-dependently in rat and mouse plasma, and the amount of YPD-29B generated was close to the amount by which IMMH-010 decreased. These data showed that IMMH-010 exhibited different stability in primate (human and monkey) plasma and rodent (rat and mouse) plasma. Moreover, IMMH-010 was quickly transformed to the active metabolite YPD-29B in rodent plasma, whereas IMMH-010 remained relatively stable in primate plasma.

### 3.3. Metabolism of IMMH-010 in Liver S9 Homogenate Fractions

**Figure 4:**
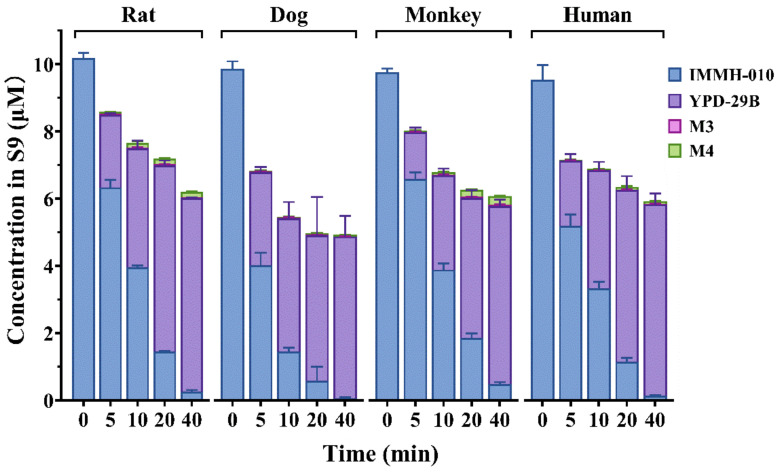
IMMH-010 metabolism in rat, dog, monkey, and human liver S9 fractions. IMMH-010 (10 μM) was incubated with liver S9 homogenate protein (0.5 mg protein/mL) and an NADPH regenerating system in a final volume of 0.2 mL Tris-HCl buffer (50 mM, pH 7.4) containing 5 mM MgCl_2_. IMMH-010 metabolism in the liver has interspecies similarities. Data are expressed as mean ± SD.

We studied the in vitro transformation of prodrug IMMH-010 in liver S9 homogenate fractions of human, monkey, dog, and rat. IMMH-010 and all the metabolites, including YPD-29B, M3, and M4, were detected in the incubation mixtures. The transformation of IMMH-010 to YPD-29B occurred quickly in a time-dependent manner in human, monkey, dog, and rat liver S9 fractions without obvious differences (Figure 4). YPD-29B was the main metabolite, and the amounts of the two other metabolites were small (<3%). The transformation was complete within 40 min in all four groups. The t_1/2_ values of IMMH-010 in human, monkey, dog, and rat liver S9 fractions were 6.66, 8.28, 4.90, and 7.10 min, respectively. The corresponding estimated CL_hep_ values of IMMH-010 were 18.2, 37.2, 36.4, and 58.4 mL/min/kg, respectively, accounting for 83.4–91.0% of hepatic blood flow. These data showed that IMMH-010 metabolism in the liver had interspecies similarities, which was different from IMMH-010 metabolism in plasma.

### 3.4. Comparison of IMMH-010 Metabolism in Liver and Intestinal S9 Fractions and Microsomes

IMMH-010 is an orally administered prodrug. Before the oral administrated prodrug reaches the systemic circulation, first-pass metabolism may occur in the intestine, followed by the portal blood and liver. To estimate the relative contribution of the intestine and liver to the first-pass bioactivation, the IMMH-010 metabolism was measured in rat liver and intestinal S9 fractions and in human liver and intestinal microsomes. The positive control, midazolam, a CYP substrate, was metabolized to 1-hydroxymidazolam in the presence of NADPH in the rat and human liver and intestine incubations. Similar to the literature [13,14], the 1-hydroxymidazolam formation rates in rat liver S9 fractions and HLM were 1.3- and 3.7-fold higher than those for the intestine fractions and microsomes, respectively, indicating that the incubation systems were reliable.

**Figure 5:**
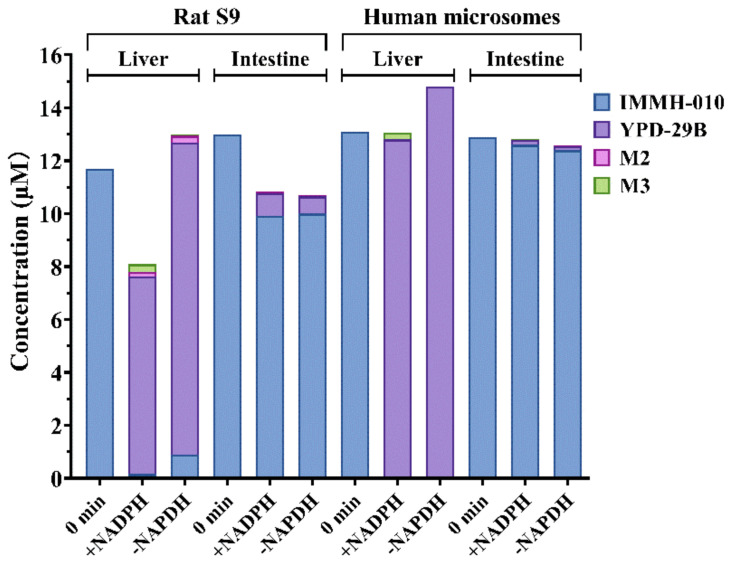
IMMH-010 metabolism in liver and intestine S9 fractions and microsomes. IMMH-010 (10 μM) was incubated with rat liver and intestinal S9 homogenate protein (1 mg protein/mL) and with human liver and intestinal microsomes (0.2 mg protein/mL) in a final volume of 0.2 mL Tris-HCl buffer (50 mM, pH 7.4) containing 5 mM MgCl_2_. The incubations were performed in duplicate in the presence and absence of an NADPH regenerating system.

After incubating for 20 min, the transformation ratios of IMMH-010 to YPD-29B in rat liver and intestinal S9 fractions were 63.8 and 6.7%, whereas the transformation ratios in human liver and intestinal microsomes were 97.7 and 1.5%, respectively (Figure 5). The transformation of IMMH-010 to YPD-29B was unrelated to the presence of NADPH. These data demonstrated that the transformation ratio of IMMH-010 to YPD-29B in the liver was much higher than in the intestine. In addition, traces of M2 and M3 (transformation ratio <2%) were detected in human liver and intestinal microsomes after incubation, and their generation was NADPH-dependent, suggesting the involvement of CYPs in IMMH-010 metabolism. Thus, the intestine may not be an important site for IMMH-010 hydrolysis in rats and humans.

### 3.5. Identification of Metabolizing Enzymes

**Figure 6:**
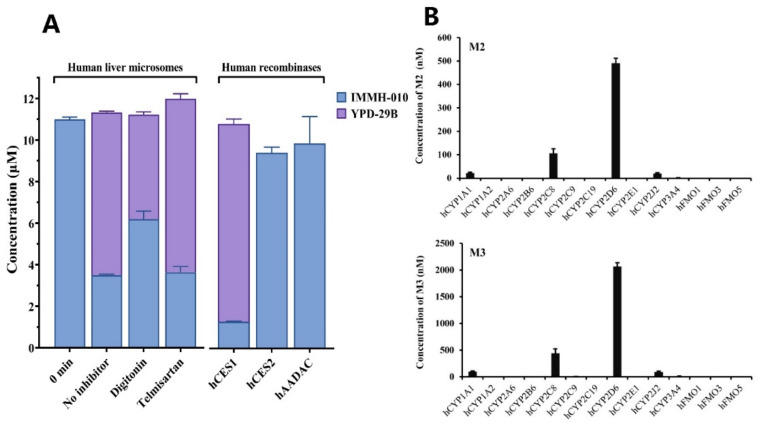
Effects of various human esterases, CYPs, and FMOs on IMMH-010 metabolism. (**A**), Effects of esterases on IMMH-010 metabolism. IMMH-010 (10 µM) was incubated with HLM (0.2 mg/mL) for 15 min at 37 °C in the presence of chemical inhibitors (left). The selective CES1 and CES2 inhibitors were digitonin (100 μM) and telmisartan (50 μM). IMMH-010 (10 µM) was incubated individually with recombinant human CES1, CES2, and AADAC (0.1 mg protein/mL) at 37 °C for 15 min (right). (**B**), Effects of CYPs and FMOs on IMMH-010 metabolism. IMMH-010 (10 µM) was incubated individually with 50 pmol of recombinant human CYPs and FMOs at 37 °C for 30 min in the presence of an NADPH regenerating system. Data are expressed as mean ± SD.

We identified the esterase involved in IMMH-010 hydrolysis to form YPD-29B in HLM. Prodrug IMMH-010 was quickly transformed to active metabolite YPD-29B in rodent plasma, but IMMH-010 remained stable in primate plasma. Consistently, CES activity is observed in rodent plasma but not primate plasma. Therefore, digitonin and telmisartan were used as selective inhibitors of CES1 and CES2, respectively, and were added separately to the mixture of HLM and IMMH-010. After the incubation, digitonin (100 μM) inhibited the formation of YPD-29B by 35.8% compared with the no inhibitor group, whereas the addition of telmisartan (50 μM) did not inhibit the formation of YPD-29B (Figure 6A). This result indicates that digitonin inhibited the activity of CES1 and interrupted IMMH-010 hydrolysis, and thus CES1 was probably involved in IMMH-010 hydrolysis.

To further examine the role of esterases in IMMH-010 metabolism, we investigated IMMH-010 hydrolysis using recombinant human CES1, CES2, and AADAC. Lidocaine, CPT-11, and phenacetin, which are the probe substrates for CES1, CES2, and AADAC, are metabolized to xylidine, SN-38, and phenetidine by the corresponding esterase, respectively. This confirmed the hydrolase activities of these recombinant enzymes. After incubating IMMH-010 (10 μM) with human CES1, CES2, and AADAC (0.1 mg/mL) separately for 15 min, the remaining amounts of IMMH-010 were 12.9%, 94.2%, and 98.7%, respectively. Furthermore, in the CES1 group, the amount of YPD-29B was equivalent to the transformation of 95.3% of IMMH-010. These results showed that IMMH-010 is converted to YPD-29B by CES1.

To understand the roles of NADPH-dependent enzymes in IMMH-010 metabolism, IMMH-010 was incubated with various human CYPs and FMOs. CYP2D6 showed the highest metabolic activity for the formation of M2 and M3, and CYP2C8, CYP1A1, and CYP2J2 were partially involved. M4 was not detected in any of the CYP and FMO incubations (Figure 6). Therefore, the other metabolizing enzymes responsible for M4 formation remain to be discovered.

### 3.6. PK/PD Study of IMMH-010 in Mice

In vivo antitumor activities of IMMH-010 were evaluated in C57BL/6 mice bearing mouse melanoma and colon carcinoma xenografts (Table 1). CTX reached TGI of 90% in both xenograft models. In the B16F10 model and MC38 model, treatment with anti-PD-1 antibody (10 mg/kg) resulted in 68% and 49% TGI, respectively. After oral administration of IMMH-010 maleate once a day for 19 days, significant reductions in tumor growth were observed in both models without weight loss. In the B16F10 model, statistically significant TGI was observed at 2.5 mg/kg (45% TGI, *p* < 0.05 vs. vehicle, *n* = 10) with maximal tumor stasis occurring at doses of 10 mg/kg (55% TGI, *p* < 0.001, *n* = 10). Significant TGI was also seen in the MC38 model at doses of 5 mg/kg (TGI = 75%, *p* < 0.001, *n* = 10) and 10 mg/kg (TGI = 57%, *p* < 0.01, *n* = 10).

**Figure 7:**
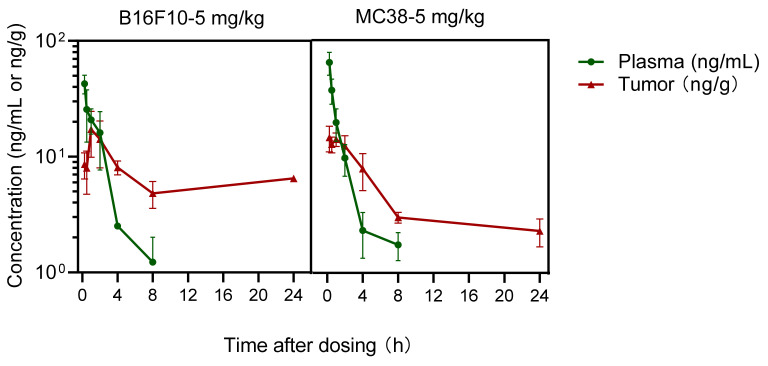
Mean plasma and tumor concentration-time profiles of active metabolite YPD-29B in B16F10 melanoma and MC38 colon cancer xenograft mice after the last oral administration of IMMH-010 maleate at a dose of 5 mg/kg for 19 days (*n* = 4). Data are expressed as mean ± SD.

The concentrations of prodrug IMMH-010 and active metabolite YPD29B were also measured in the plasma and tumor tissue of B16F10 melanoma and MC38 colon cancer xenograft mice. After the last oral administration of IMMH-010 maleate (5 mg/kg), only traces of IMMH-010 (<1 ng/mL) were found in plasma and tumor tissues. In B16F10 melanoma and MC38 colon cancer xenograft mice, active hydrolyzed metabolite YPD-29B appeared rapidly in plasma (Figure 7). The mean peak concentrations (C_max_) of YPD-29B were 42.65 and 64.43 ng/mL, respectively, occurring at a mean time of 15 min for both. The average elimination half-life (t_1/2β_) values of YPD-29B in B16F10 melanoma and MC38 colon cancer xenograft mice were 1.61 and 1.76 h, respectively, and the areas under the plasma concentration versus time curve (AUC) of YPD-29B in the two groups of tumor xenograft mice were similar (69.9 ng/mL∙h). The maximum concentrations of YPD-29B in the tumor were obtained 15–60 min after dosing, which was slightly delayed compared with the peak in plasma. Then, YPD-29B was eliminated slowly in tumors of B16F10 melanoma and MC38 colon cancer xenograft mice, with mean t_1/2β_ values of 12.37 and 44.99 h, respectively. Therefore, YPD-29B had a higher exposure in tumors, and the tissue/plasma ratios (AUC_tumor_/AUC_plasma_) were 2.1 and 2.4, respectively.

### 3.7. PK Study of IMMH-010 in Monkeys

**Figure 8:**
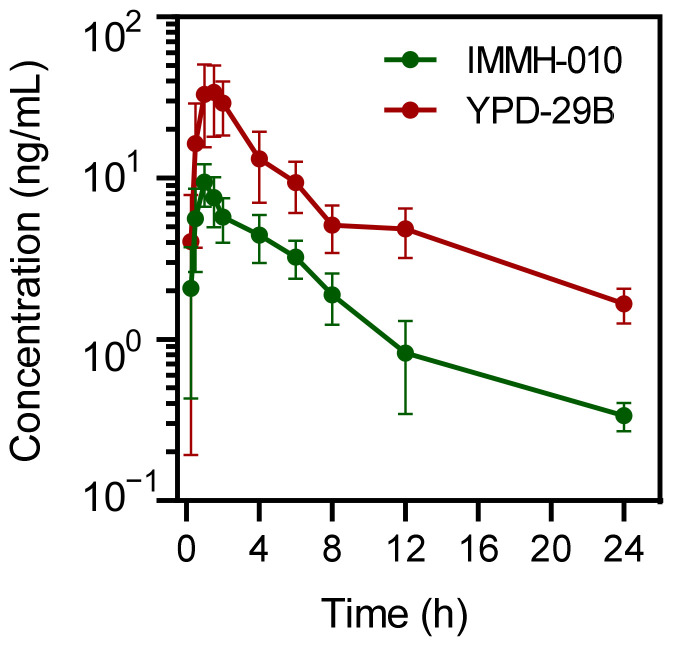
Mean plasma concentration-time profiles of IMMH-010 and active metabolite YPD-29B in male monkeys after oral administration of IMMH-010 maleate at a dose of 5 mg/kg (*n* = 4). Data are expressed as mean ± SD.

Because the in vitro studies suggested that there was a large difference in IMMH-010 metabolism in primate and rodent plasma, we measured the pharmacokinetic differences between rodents and primates to provide a reference for the clinical research. We evaluated the PK of IMMH-010 in male cynomolgus monkeys. After a single oral administration of IMMH-010 maleate of 5 mg/kg, the average C_max_ values of IMMH-010 and YPD-29B were 9.46 and 35.5 ng/mL, respectively. The observed times to peak IMMH-010 and YPD-29B concentration were within 1.5 h of administration. The mean t_1/2β_ values of plasma IMMH-010 and YPD-29B were 5.16 and 9.00 h, respectively. The AUC values of IMMH-010 and YPD-29B were 47.9 and 186 ng/mL∙h, respectively. The molar AUC ratio of YPD-29B to IMMH-010 was 4.17 after oral administration of 5 mg/kg IMMH-010 maleate (Figure 8). Therefore, after absorption, although IMMH-010 was not completely converted to YPD-29B, IMMH-010 underwent rapid biotransformation and the conversion rate was high in monkeys.

## 4. Discussion

Blocking the PD-1/PD-L1 interaction is a powerful strategy in cancer immunotherapy and much research has focused on developing effective PD-1/PD-L1 inhibitors. IMMH-010, which was designed as a prodrug of potent PD-1/PD-L1 inhibitor YPD-29B, is currently in a phase I clinical trial. A pharmacokinetic study of IMMH-010 helped to reveal the mode of action of this type of PD-1/PD-L1 inhibitor and provided useful information for drug development and clinical applications. In the present study, we analyzed the metabolites of IMMH-010 in vivo and in vitro and confirmed that YPD-29B is the main metabolite of IMMH-010. We also used various recombinant esterases to hydrolyze IMMH-010 to YPD-29B and found that IMMH-010 hydrolysis is catalyzed by CES1. In summary, our metabolism research showed that our prodrug strategy worked as expected.

We explored IMMH-010 metabolism in detail, including the major metabolic sites and the species variation. There are species and tissue differences in esterase activities [11,15]. These differences determined which tissue is the primary metabolic site for IMMH-010 and the interspecies differences in IMMH-010 metabolism. The major classes of esterases involved in drug metabolism include butyrylcholinesterases, CESs, paraoxonases, and AADACs [16,17,18,19,20]. The expressions of these esterases have been reported. Butyrylcholinesterase activity is mainly observed in the plasma of mice, rats, dogs, monkeys, and humans, but the activity is low in rats [11]. CES activity is observed in the blood of mice and rats but not in the blood of dogs, monkeys, or humans. CES is also present in the liver and intestine of these species. Mouse, rat, and monkey intestine show higher CES activity than the human intestine. Paraoxonase activity is mainly found in the plasma and liver of mice, rats, dogs, monkeys, and humans, but it is lower in monkey plasma and liver. AADAC is expressed in the liver and gastrointestinal tract.

In the present study, human, monkey, rat, and mouse plasma were used to study the in vitro metabolism of IMMH-010. IMMH-010 was found to be rapidly metabolized in rat and mouse plasma, but it was stable in monkey and human plasma. This result is consistent with the distribution of esterases in various species; CES activity is observed in the blood of mice and rats, but not in the blood of dogs, monkeys, or humans. Thus, this result also indicated that IMMH-010 was mainly hydrolyzed to active metabolite YPD-29B by CES1.

CES is a member of the serine hydrolase superfamily. Human CES is classified into five families: CES1, CES2, CES3, CES4A, and CES5A. CES1 and CES2 hydrolyze a number of clinically used prodrugs and drugs [21]. Except for oxybutinin, CES1 substrates have a large acyl group and small alcohol group, whereas a small acyl group and large alcohol group are common features of CES2 substrates [18]. AADAC is a microsomal serine esterase. Similar to CES2, AADAC prefers substrates with a small acyl group and large alcohol group. We performed inhibition studies by using inhibitors to estimate the contribution of CES1 and CES2 to IMMH-010 hydrolysis in HLM. Shimizu et al. [22] reported that digitonin is a specific inhibitor of CES1, but due to the interference of certain proteins, its inhibitory effect in HLM is weak, and telmisartan is a strong inhibitor of CES2 in both HLM and recombinants. IMMH-010 contains a large acyl group and a small alcohol group. We observed that digitonin (100 μM) inhibited the formation of YPD-29B by 35.8% in HLM, whereas telmisartan (50 μM) showed no inhibition, indicating that CES1 may be involved in IMMH-010 hydrolysis. To evaluate further the contribution of CES1, CES2, and AADAC to IMMH-010 metabolism, recombinant human CES1, CES2, and AADAC were used. The results confirmed that CES1 rather than CES2 and AADAC catalyzed IMMH-010 hydrolysis.

We compared the relative contribution and species difference of the intestine and liver to IMMH-010 bioactivation. Because liver S9 fractions are better than hepatocytes for predicting the hepatic metabolic clearance of ester prodrugs [23], we compared IMMH-010 metabolism in human, monkey, dog, and rat liver S9 fractions. Furthermore, IMMH-010 hydrolysis in rat liver and intestinal S9 fractions and in human liver and intestinal microsomes was compared. The results showed that IMMH-010 was hydrolyzed rapidly in all species in a similar manner, and the intestine did not contribute much to the first-pass metabolism of IMMH-010. Consistently, CES1 is predominantly expressed in the liver of all species, whereas CES2 is expressed in the gastrointestinal tract. Thus, CES1, but not CES2, was further considered to have the major role in IMMH-010 bioactivation.

Because IMMH-010 bioactivation in humans and monkeys was similar in our in vitro studies, we speculated that the PK of IMMH-010 in monkeys might be similar to that in humans. Considering the transformation ratio of IMMH-010 to YPD-29B in human liver S9 fractions was slightly higher than that of monkeys (53.5% vs. 42.6%), the exposure of YPD-29B in humans may be higher than that in monkeys.

B16F10 melanoma cells and MC38 colon cells are widely used in anti-PD-L1 cancer immunotherapy because they express PD-L1 [24]. In this study, B16F10 melanoma and MC38 colon tumor models were established. The antitumor activity and the PK characteristics of IMMH-010 were evaluated in both models. Significant reductions in tumor growth were observed in both models without weight loss. Therefore, IMMH-010 demonstrated robust antitumor activity. The PK of active metabolite YPD-29B in the two tumor xenograft mice models was similar. YPD-29B was eliminated slowly in tumors. The t_1/2β_ of YPD-29B in tumors was 7.6–25 times longer than that of plasma, which may be related to its receptor binding ability, resulting in higher drug exposure in tumors, which is beneficial for treatment. Compared to normal tissues, the expression and activity of drug-metabolizing enzymes are significantly dysregulated in tumor tissues [25]. The higher exposure of YPD-29B in tumors may be related to the changes in the activity of drug-metabolizing enzymes in tumor tissues and the combination of YPD-29B and PD-L1.

## 5. Conclusions

In conclusion, four metabolites of IMMH-010 were identified in this study, of which the active hydrolysis metabolite YPD-29B was the predominant metabolite. IMMH-010 was mainly catalyzed to YPD-29B by CES1 in the human liver. IMMH-010 metabolism in the liver S9 fractions of all species investigated was similar. IMMH-010 exhibited remarkable antitumor efficacy in B16F10 melanoma and MC38 colon carcinoma xenograft mouse models. The t_1/2β_ of YPD-29B in tumors was 7.6–25 times longer than that in plasma. Our results advance the understanding of the metabolism and mechanism of IMMH-010 and lay the foundations for further clinical applications.

## Figures and Tables

**Table 1:** Effects of IMMH-010 on the body weight and tumor growth in B16F10 and MC38 models after administration for 19 days.

Model	Group	Dose (mg/kg)	Number (Start/Finish)	Body Weight (g) X ± SD		Tumor Weight (g)	
				Start	Finish	X ± SD	TGI (%)
B16F10	Control		10/10	17.32 ± 0.46	21.0 ± 0.7	2.32 ± 0.85	NA
	CTX	80	10/10	16.94 ± 0.43	19.2 ± 0.8	0.23 ± 0.18 ***	90
	PD-L1 Antibody	10	10/10	16.7 ± 0.53	19.4 ± 0.9	0.74 ± 0.61 ***	68
	IMMH-010	1.25	10/10	17.09 ± 0.63	20.2 ± 1.4	1.78 ± 1.13	23
		2.5	10/10	16.86 ± 0.57	20.3 ± 1.2	1.26 ± 0.85 *	45
		5	10/10	17.09 ± 0.81	20.0 ± 1.2	1.39 ± 0.84 *	40
		10	10/10	17.15 ± 0.50	20.1 ± 1.0	1.04 ± 0.66 **	55
MC38	Control		10/10	22.0 ± 0.4	26.1 ± 1.3	1.70 ± 0.75	NA
	CTX	40	10/10	22.0 ± 0.8	24.5 ± 0.9	0.17 ± 0.10 **	90
	PD-L1 Antibody	10	10/10	22.0 ± 0.7	25.7 ± 1.7	0.87 ± 0.55 ***	49
	IMMH-010	1.25	10/10	22.2 ± 0.4	24.3 ± 2.1	1.03 ± 0.65 *	40
		2.5	10/10	22.0 ± 0.6	25.3 ± 2.3	1.13 ± 0.78	34
		5	10/10	21.9 ± 0.8	23.7 ± 1.8	0.42 ± 0.39 ***	75
		10	10/10	21.9 ± 0.7	25.0 ± 1.7	0.73 ± 0.54 **	57

NA: not applicable, SD: standard deviation, TGI: tumor growth inhibition (100 − treatment group tumor weight/vehicle group tumor weight × 100) Data are expressed as mean ± SD. (* *p* < 0.05, ** *p* < 0.01, *** *p* < 0.001, *n* = 10).

## Data Availability

All data generated or analyzed during the study appear in the submitted article.
